# Broiler genetics influences proteome profiles of normal and woody breast muscle

**DOI:** 10.1016/j.psj.2021.01.017

**Published:** 2021-01-16

**Authors:** Xue Zhang, K. Virellia To, Tessa R. Jarvis, Yan L. Campbell, Jasmine D. Hendrix, Surendranath P. Suman, Shuting Li, Daniel S. Antonelo, Wei Zhai, Jing Chen, Haining Zhu, M. Wes Schilling

**Affiliations:** ∗ Department of Food Science, Nutrition, and Health Promotion, Mississippi State University, Mississippi State 39762, USA; † Department of Animal Science, Iowa State University, Ames 50011, USA; ‡ Department of Animal and Food Sciences, University of Kentucky, Lexington 40546, USA; § Department of Animal Nutrition and Production, College of Veterinary Medicine and Animal Science, University of Sao Paulo, Pirassununga/SP 13635-900, Brazil; # Department of Poultry Science, Mississippi State University, Mississippi State 39762, USA; ‖ Proteomics Core Facility, University of Kentucky, Lexington 40506, USA

**Keywords:** woody breast myopathy, proteomics, broiler genetics, poultry

## Abstract

Wooden or woody breast (**WB**) is a myopathy of the pectoralis major in fast-growing broilers that influences the quality of breast meat and causes an economic loss in the poultry industry. The objective of this study was to evaluate growth and proteome differences between 5 genetic strains of broilers that yield WB and normal breast (**NB**) meat. Eight-week-old broilers were evaluated for the WB myopathy and divided into NB and WB groups. Differential expression of proteins was analyzed using 2-dimensional gel electrophoresis and LC-MS/MS to elucidate the mechanism behind the breast myopathy because of the genetic backgrounds of the birds. The percentages of birds with WB were 61.3, 68.8, 46.9, 45.2, and 87.5% for strains 1-5, respectively, indicating variability in WB myopathy among broiler strains. Birds from strains 1, 3, and 5 in the WB group were heavier than those in the NB group (*P* < 0.05). Woody breast meat from all strains were heavier than NB meat (*P* < 0.05). Within WB, strain 5 had a greater breast yield than strains 1, 3, and 4 (*P* < 0.0001). Woody breast from strains 2, 3, 4, and 5 had a greater breast yield than NB (*P* < 0.05). Six proteins were more abundant in NB of strain 5 than those of strains 2, 3, and 4, and these proteins were related to muscle growth, regeneration, contraction, apoptosis, and oxidative stress. Within WB, 14 proteins were differentially expressed between strain 5 and other strains, suggesting high protein synthesis, weak structural integrity, intense contraction, and oxidative stress in strain 5 birds. The differences between WB from strain 3 and strains 1, 2, and 4 were mainly glycolytic. In conclusion, protein profiles of broiler breast differed because of both broiler genetics and the presence of WB myopathy.

## Introduction

The increased demand for poultry meat since 2000 has been attributed to good nutritional value, low cost, and suitability for further processing (Petracci et al., 2015). This increased demand has contributed to the need for greater production efficiency. As a result, breeders have focused on high growth rate and breast yield hybrids, which has led to continuous improvements in broiler production (Soglia et al., 2016). Unfortunately, improvements in genetic selection have also contributed to the development of muscle abnormalities and myopathies, including “wooden or woody breast” (Petracci et al., 2015; Soglia et al., 2016).

Woody breast (**WB**) starts developing in broilers at as early as 2 wk of age. A chronic myodegradation may appear at 3 to 4 wk of age (Baltic et al., 2019). According to Sihvo et al. (2014), WB is a myopathy commonly found on the pectoralis major in which the hardened area is visibly bulging and pale. Various factors affect the development of WB, including the broiler's genotype, gender, egg incubation condition, diet, and feeding allocation (Tijare et al., 2016; Bowker and Zhuang, 2017; Meloche et al., 2018; Zhang et al., 2020a; Oviedo-Rondón et al., 2020). One factor that has been consistently associated with the incidence of WB is heavier body weight and thicker fillets (Trocino et al., 2015; Baltic et al., 2019).

Compositional differences in WB negatively impact the appearance and functional meat quality. According to Tijare et al. (2016), the altered texture of WB fillets is likely a result of the high content of connective tissue and damaged muscle fiber structure. Woody breast fillets have experienced increased muscle fiber degeneration and regeneration, necrosis, fiber size variability, lipid infiltration, increased fibrosis, and inflammatory cell invasion (Bowker and Zhuang, 2017). Woody breast meat has reduced water-holding capacity when compared with normal breast (**NB**) (Bowker and Zhuang, 2017). Soglia et al. (2016) suggested that the proportion and mobility of the extra-myofibrillar water fraction within the muscle increases in the WB condition, which decreases the ability of the muscle to retain water. Even though this quality issue does not impose a food safety risk, its unappealing appearance and impaired nutritional quality (higher in fat and collagen, poor water-holding capacity and texture) have a negative economic impact on poultry companies (Soglia et al., 2016; Bowker and Zhuang, 2017; Baltic et al., 2019) because WB is downgraded or even discarded in more severe cases.

Proteomic techniques have been applied to study the muscle protein profiles of livestock with different traits or with different genetic origins (Yu et al., 2016; Paredi et al., 2017; Nair et al., 2018), poultry meat quality, and defects (Kuttappan et al., 2017a; Cai et al., 2018). In our previous studies, proteomic profiles of WB and NB from 5 different broiler strains were characterized, and the proteins that were found in abundance in WB were related to oxidative stress, structural, and transport proteins in which the 4 most consistently present proteins (annexin A2, apolipoprotein A-1, cofilin-2, and heat shock protein beat-1) were major contributors to the development of WB (Zhang et al., 2020a). Research has been conducted to compare proteome profiles of chicken breast, and 11 proteins were differentially abundant between 3 Thai local chicken breeds of the same age (Zanetti et al., 2009, 2011). However, little is known about the impact of broiler genetics on WB development and incidence. The objective of this study was to evaluate how genetics affected birds' growth and altered proteomic profiles of WB and NB meat, as well as identify differentially expressed proteins that could serve as potential biomarkers for the WB myopathy in 5 genetically different broiler strains.

## Materials and methods

### Eggs and Broilers

This study was approved by the Institutional Animal Care and Use Committee at Mississippi State University (approval # IACUC-16-542A). Eggs were procured from 5 commercial breeder hens (strains 1-5) that were 30 wk old. All eggs were collected within the same period and placed in a single-stage incubator (Chick Master Incubator Co, Medina, OH). The incubator was set at 37.5°C and 55% relative humidity from day 1 to 18 of incubation. The eggs were transferred into the hatcher on day 18. The hatcher was set at 36.7°C with 60% relative humidity till day 21 of incubation when the chicks were hatched. Eggs were candled on day 11, and dead and infertile eggs were removed. Eggs were transferred to a hatcher (Chick Master Incubator Co.) on day 18 of incubation. On day 21, a total of 640 (128 birds/strain) newly hatched chicks were transferred to the Mississippi State University Poultry Farm. The farm was divided into 8 blocks, and 128 chicks of each strain were randomly assigned to 8 pens (16 birds/pen/block, 0.0846 m^2^/bird). These newly hatched chicks weighed 39.7, 39.7, 41.8, 39.4, and 40.2 g for strains 1-5, respectively (Zhang et al., 2020b). Chicks were fed with a control diet that was formulated to meet the highest recommended digestible amino acids (Zhang et al., 2020b). These 5 commercially used broiler strains differ in genetic background. Strains 1 and 2, strain 3, and strains 4 and 5 were from 3 different breeding companies. In the chicken house, each pen was equipped with one hanging feeder and 3 nipple drinkers. Water and feed were provided on an ad libitum basis. The birds received a 24 L:0 D photoperiod from day 0 to 7 and a 20 L:4 D photoperiod from day 8 to 60. The temperature of the house was 34°C on the day of hatch, gradually decreased to 31°C on day 7 of age, 27°C on day 14, 24°C on day 21, 21°C on day 28, 19°C on day 35, 18°C on day 42, and stayed at 18°C thereafter.

### Processing and Sampling

At 8 wk of age, live male birds were evaluated for WB myopathy by manual palpation. For each strain of birds, 4 birds (n = 1 bird/pen) with NB were selected from blocks 1-4, and 4 birds (n = 1 bird/pen) with WB were selected from blocks 5-8. After euthanizing broilers with CO_2_ gas, 5-10 g of muscle from the cranial portion of the breast was collected and snap-frozen in liquid nitrogen. The proteomic profiles of broiler breast muscle were evaluated.

After 8 wk, broilers (n = 4 birds/pen, 160 birds in total) were randomly selected, weighed, and tagged for processing. After 14 h fasting, birds were processed in the processing plant at the Mississippi State University Poultry Farm. Body weight, carcass weight, and pH_15min_ of the breast were immediately measured after processing. Part weights, including breast, tender, wings, drumsticks, and thighs, were weighed after carcasses were chilled for 4 h in ice water. The WB myopathy and white striping (**WS**) were evaluated at 24 h postmortem according to Tijare et al. (2016), where 0 = normal, 1 = slight, 2 = moderate, and 3 = severe. Breast samples were grouped into NB with scores of 0 and 1 and WB with scores of 2 and 3. Slight WB was considered as NB because manual palpation on live birds did not differentiate these 2 groups. Breast pH and surface color were also evaluated at 24 h postmortem. Breast samples were stored at −18°C for 6 to 8 wk before cooking loss and shear force analysis.

### Physicochemical Properties of Broiler Breast Meat

#### pH Measurement

The pH values of the breast muscle (n = 32/strain) were measured using a pH meter (Model Accumet 61a; Fisher Scientific, Hampton, NH) with a penetrating pH probe (Model FlexipHet SS Penetration tip; Cole Palmer, Vernon Hills, IL). The pH was measured on the processing line at 15 min and 24 h postmortem by inserting the pH probe in the cranial part at 2.5 cm below the top of the fillet.

#### Color Measurement

The color of NB and WB samples were determined at the surface (skin side) of the breast muscle (n = 32/strain) at 3 different locations (cranial, middle, and caudal) at 24 h postmortem. Color was evaluated using a portable, reflected-color spectrophotometer (MiniScan EZ 4500L; HunterLab, Reston, VA) with a 31.8-mm port size, a 10° standard observer, and a D65 illuminant. The color attributes were expressed as CIE *L∗* (lightness), *a∗* (redness), and *b∗* (yellowness). Hue angle [tan^−1^(*b*∗/*a*∗)] and chroma [(*a*∗^2^ + *b*∗^2^)^1/2^] were calculated (AMSA, 2012).

#### Cooking Loss and Warner-Bratzler Shear Force Determination

Half of the chicken breast samples (2 birds/pen, n = 16 in total) were thawed overnight at 4°C. Breast samples were weighed (initial weight) and then baked in a preheated oven (Viking, Greenwood, MS) at 177°C to a final internal temperature of 77°C. After cooking, the breast samples were cooled to room temperature (22 ± 2°C) and weighed again (final weight). Cooking loss of breast samples was reported as a percentage and calculated as [(initial weight − final weight)/(initial weight)] × 100%.

For Warner-Bratzler shear force analysis, 6 adjacent 1 cm (width) × 1 cm (thickness) × 2 cm (length) strips were cut from the cranial part of the breast sample in a direction that was parallel to the muscle fibers. Samples were sheared perpendicular to the muscle fibers using a Warner-Bratzler shear attachment that is mounted to an Instron Universal Testing Center (Model 3300; Instron, Norwood, MA). The shear force was reported as the maximum peak force (N) that was required to cut through the chicken breast strips (Schilling et al., 2012).

### Protein Extraction

Proteomic analysis was carried out on the NB (n = 3/strain) and WB (n = 3/strain) tissues as previously described (Zhang et al., 2020a). Frozen breast tissue (1-2 g) was ground in liquid nitrogen, and 200 mg of ground tissue was homogenized for 30 s using a homogenizer (FSH 500; Thermo Fisher Scientific, Waltham, MA) in 1.0 mL of ice-cold buffer containing 8.3 M urea, 2 M thiourea, 2% CHAPS 3-((3-cholamidopropyl) dimethylammonio)-1-propanesulfonate, and 1% dithiothreitol (**DTT**). The homogenate was further mixed for 2 h in ice water on a magnetic stirrer followed by centrifugation at 18,000 *g* for 30 min at 4°C. The protein concentration in the supernatant was determined using the Bradford Assay (Bio-Rad, Hercules, CA).

### Two-Dimensional Gel Electrophoresis

**Figure 1:**
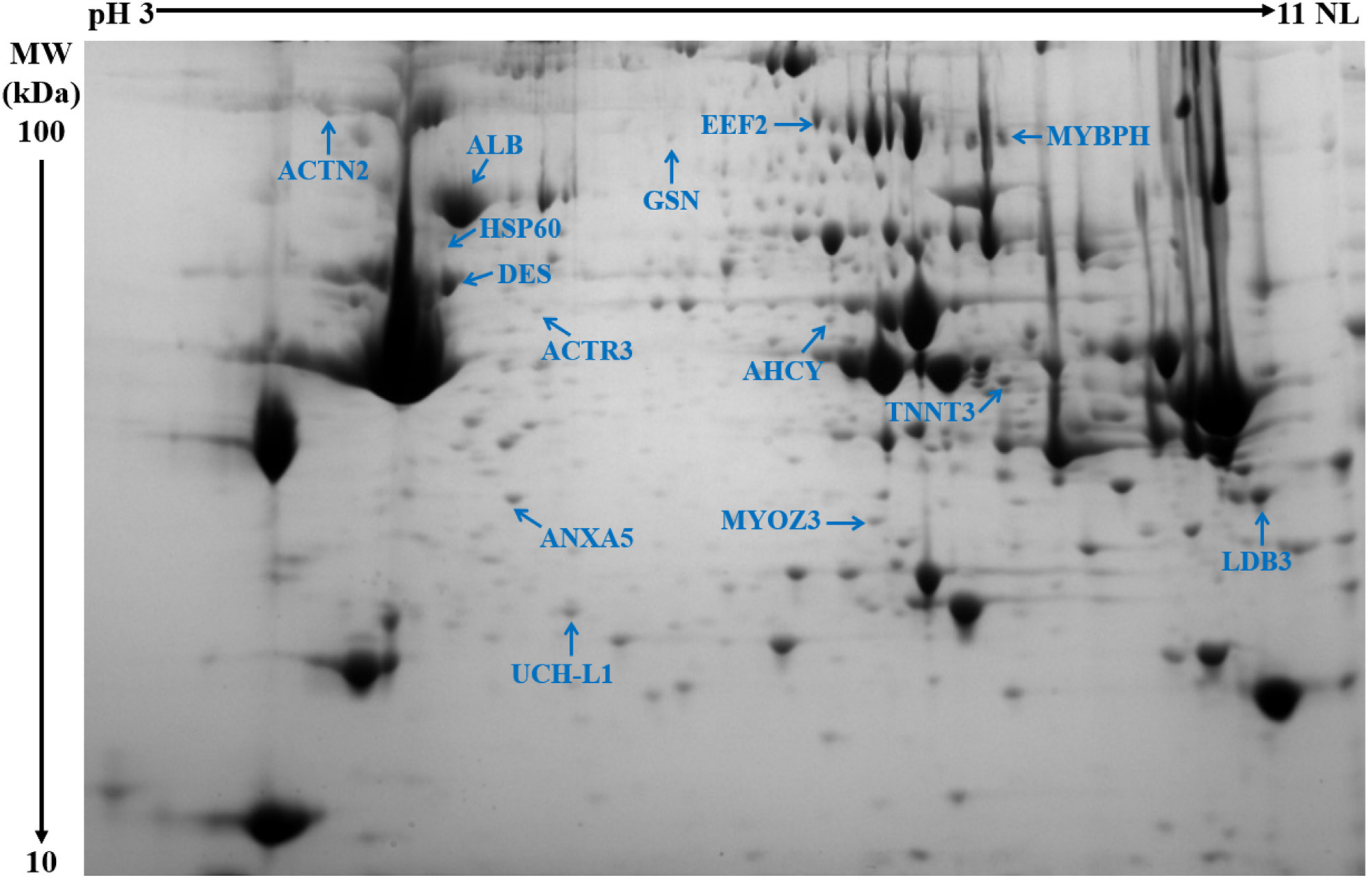
A representative 2-dimensional gel electrophoresis image of whole muscle proteome from woody breast of strain 5 separated using an immobilized non-linear (NL) pH gradient (IPG) pH 3-11 strip (11 cm) and a 12.5% Criterion precast Tris-HCl gel. The protein loading was 500 μg, and the gel was stained with brilliant blue G-colloidal solution. Abbreviations: ACTN2, alpha-actinin 2; ACTR3, actin-related protein 3; AHCY, adenosylhomocysteinase; ANXA5, annexin A5; ALB, serum albumin precursor; DES, desmin; EEF2, elongation factor 2; GSN, gelsolin, HSP60, 60 kDa heat shock protein (mitochondrial), LDB3, LIM domain-binding protein 3; MYBPH, myosin-binding protein H; MYOZ3, myozenin-3; TNNT3, troponin T, fast skeletal muscle isoform; UCH-L1, ubiquitin carboxyl-terminal hydrolase.

The protein extract (500 μg) was included in a Destreak rehydration buffer (GE Healthcare, Chicago, IL) with the addition of 1% DTT and 0.5% carrier ampholytes (GE Healthcare, Chicago, IL). The mixture was centrifuged at 14,000 *g* for 5 min, and the supernatant was applied onto immobilized non-linear pH gradient (**IPG**) strips (pH 3 to 11, 11 cm; GE Healthcare) and covered with 2 mL mineral oil. The first-dimension isoelectric focusing was performed using a Protean Isoelectric Focusing system (Bio-Rad, Hercules, CA). Gels were passively rehydrated for 12 h, and subsequently applied voltage to reach a cumulative 35 kV h. The IPG strips were equilibrated for 15 min in equilibration buffer I (Bio-Rad, Hercules, CA) and another 15 min in Equilibration buffer II (Bio-Rad, Hercules, CA). In the second dimension, proteins were resolved on 12.5% Criterion Precast gels (Bio-Rad, Hercules, CA) in tris-glycine electrophoresis buffer. Gels were stained in Brilliant Blue G-Colloidal solution (Sigma-Aldrich, Milwaukee, WI) and de-stained in 25% methanol (Fisher Scientific, Pittsburgh, PA). Two gels were produced for each protein extract sample. Figure 1 contains a representative gel image from strain 5 WB tissue.

### Gel Visualization and Image Acquisition

Gel images were acquired using a VersaDoc Model 3000 imaging system (Bio-Rad) and were analyzed using PDQuest software (Bio-Rad). Image analysis was performed as previously described (Zhang et al., 2020c). Protein spots from the NB (n = 6 gels) and WB (n = 6 gels) gel images were detected and analyzed using qualitative, quantitative, and statistical modes. Protein spots in each comparison were considered differentially abundant when they exhibited a 2.0-fold or more intensity difference that was associated with a 5% statistical significance (*P* < 0.05) in the Student's *t* test.

### Protein Identification by Mass Spectrometry

The protein spots that were excised from gels were subjected to DTT reduction, iodoacetamide alkylation, and in-gel trypsin digestion according to Desai et al. (2014) and analyzed using liquid chromatography (**LC**)-tandem mass spectrometry (**MS/MS**) as previously described (Zhang et al., 2020a). The LC-MS/MS analysis was performed using a Linear Trap Quadrupole (**LTQ**) Orbitrap Velos mass spectrometer (Thermo Fisher Scientific, Waltham, MA) coupled with an Eksigent cHiPLC-nanoflex system (Eksigent, Dublin, CA) through a nano-electrospray ionization source.

The LC-MS/MS data were submitted to a local MASCOT server for MS/MS protein identification via Proteome Discoverer (version 1.3; Thermo Fisher Scientific, Waltham, MA) against a custom database of *Gallus gallus* (Chicken) proteins downloaded from the National Center for Biotechnology Information as previously described (Zhang et al., 2020a).

### Statistical Analysis

A randomized complete block design was used to select birds from the chicken house and process birds. After evaluating woody breast myopathy, the birds were divided into NB and WB groups. The differences among 5 genetic strains of birds with respect to body weight (**BW**), part weights, part percentage (the ratio of part weight to BW), pH, color, cooking loss, and shear force were analyzed using one-way analysis of variance (ANOVA) within either NB or WB group (*P* < 0.05; SAS version 9.4, NC). As samples from 5 strains of broilers within NB or WB group were unbalanced and strain 5 had only 4 samples in NB group and 28 samples in WB group, no matter if the analysis from the model was significant between treatments, the means were separated using MEANS statement that was adjusted by Tukey's Honestly Significant Difference (**HSD**) test (*P* < 0.05). The difference of each attribute between NB and WB within each strain was also compared using MEANS statement that was adjusted by Tukey's HSD test (*P* < 0.05). The correlations between WB and WS, pH_24h_, BW, breast weight, and breast yield were analyzed using Spearman's rank correlation within each strain.

## Results

### Weights

For each broiler strain, birds were processed and grouped into NB or WB after grading the breast myopathy at 24 h postmortem. There were 61.3, 68.8, 46.9, 45.2, and 87.5% birds that had WB myopathy for strains 1-5, respectively (Table 1). Within NB group, strains 3 and 4 had greater live BW (*P* = 0.0003) than strains 2 and 5. There were differences in carcass (*P* = 0.001), wing (*P* < 0.0001), thigh (*P* = 0.0003) and drumstick (*P* < 0.0001) weights among strains (Table 1). Mean separation with Tukey's HSD indicated that strain 5 yielded less breast meat (*P* < 0.05) than other strains; however, the overall *P*-value (0.091) of the model was not significant, which was likely due to the small sample size of strain 5 NB (n = 4). Expressed as a percentage of BW, however, there were only differences in breast (*P* = 0.047) and tender (*P* = 0.001) between strains where strain 2 had higher breast and tender yields than strain 4.

**Table 1:** Processing weights and yields of 5 strains of broilers with normal and woody breast on day 56.

Strain (N)^1^	Processing weight (g)							Processing yield (% of BW)					
	BW (g)	Carcass (g)	Breast (g)	Tender (g)	Wing (g)	Thigh (g)	Drumstick (g)	Carcass (%)	Breast (%)	Tender (%)	Wing (%)	Thigh (%)	Drumstick (%)
Birds with Normal Breast													
Strain 1 (12)	3,748^a,b,^∗	2,698^a,b,^∗	744^a,b,^∗	166	282^a,b^	462^a,b^	342^b,c^	72.0	19.8^a,b^	4.41^a,b^	7.53	12.3	9.16
Strain 2 (10)	3,580^b^	2,606^a,b^	745^a,b,^∗	174	264^b^	424^b^	325^c^	72.9	20.9^a,^∗	4.86^a,^∗	7.42	11.8	9.12
Strain 3 (17)	4,027^a,^∗	2,899^a,^∗	776^a,^∗	179∗	303^a,^∗	511^a,^∗	377^a,b,^∗	72.2	19.3^a,b,^∗	4.45^a,b,^∗	7.53	12.7	9.36
Strain 4 (17)	4,042^a^	2,868^a^	750^a,b,^∗	163	302^a^	504^a^	390^a^	71.0	18.6^b,^∗	4.05^b^	7.50	12.5	9.64
Strain 5 (4)	3,252^b,^∗	2,325^b,^∗	625^b,^∗	159	248^b,^∗	392^b,^∗	291^c,^∗	71.4∗	19.1^a,b,^∗	4.83^a^	7.65	12.1	8.98
SEM	123	89.2	31.4	7.61	8.45	20.3	13.7	0.660	0.609	0.164	0.145	0.311	0.210
*P*-value	0.0003	0.001	0.091^2^	0.212	<0.0001	0.0003	<0.0001	0.183	0.047	0.001	0.926	0.172	0.140
Birds with Woody Breast													
Strain 1 (19)	4,119^a,b,^∗	2,957^a,b,^∗	863∗	175	302^a,b^	491^b,c^	367^a,b,c^	71.8^b^	20.9^b,c^	4.24^a,b^	7.30	11.9^b^	8.88^c^
Strain 2 (22)	3,739^b^	2,738^b^	836∗	166	273^b^	449^c^	329^c^	73.2^a,b^	22.3^a,b,^∗	4.44^a,^∗	7.31	12.0^b^	8.79^c^
Strain 3 (15)	4,399^a,^∗	3,228^a,^∗	910∗	179	333^a,^∗	575^a,^∗	423^a,^∗	73.4^a,b^	20.7^c,^∗	4.08^a,b,^∗	7.56	13.1^a^	9.62^a^
Strain 4 (14)	4,248^a^	3,049^a,b^	841∗	165	308^a,b^	534^a,b^	410^a,b^	71.7^b^	19.8^c,^∗	3.88^b^	7.24	12.5^a,b^	9.60^a,b^
Strain 5 (28)	4,029^a,b,^∗	2,964^a,b,^∗	948∗	173	291^b,^∗	479^b,c,^∗	363^b,c,^∗	73.6^a,^∗	23.5^a,^∗	4.33^a,b^	7.24	11.9^b^	9.01^b,c^
SEM	123	93.4	32.8	6.57	9.78	18.2	14.1	0.391	0.400	0.126	0.091	0.216	0.159
*P*-value	0.004	0.010	0.041^3^	0.533	0.001	<0.0001	<0.0001	0.0005	<0.0001	0.027	0.125	0.0005	0.0003

^a-c^Means with the same letter are not different due to strain within normal or woody breast bird groups.

Means without a ‘∗’ are not different between normal and woody breast birds within each strain.

1 For each strain, 32 birds were selected for processing. Strains 1 and 4 had 31 samples due to the missing tags in the processing.

2 ANOVA test did not show significance due to the very small sample size of strain 5 in NB group (n = 4) that does not provide enough power to show significant results. Power is the probability of rejecting the null hypothesis when the alternative hypothesis is true.

3 ANOVA test showed significant results but mean separation did not because the very large sample size of strain 5 in WB group (n = 28) that provides too much power.

Within WB, strains 3 and 4 had greater BW (*P* = 0.0042) than strain 2. There were differences in carcass (*P* = 0.010), breast (*P* = 0.040), wing (*P* = 0.001), thigh (*P* < 0.0001) and drumstick (*P* < 0.0001) weights among strains (Table 1). However, mean separation did not indicate differences in breast weight, which was likely due to the large sample size of strain 5, which created too much power in variance analysis. Expressed as a percentage of BW, unlike NB birds, there were difference in carcass and parts among strains (*P* < 0.05), with the exception of the wing. Within WB, strain 5 had a greater carcass yield than strains 1 and 4 (*P* = 0.005). Strain 5 had a greater breast yield than strains 1, 3, and 4, and strain 2 had a greater breast yield than strains 3 and 4 (*P* < 0.0001). Strain 2 had a greater tender yield than strain 4 (*P* = 0.027), and there was no difference in strains 1, 3, 4 and 5 (*P* > 0.05). Strain 3 had a greater thigh yield than strains 1, 2, and 5 (*P* = 0.0005). Strain 3 also had greater drumstick yield than strains 1 and 2 (*P* = 0.0003) (Table 1).

Within each strain, strains 3 and 5 with WB had greater body, carcass, breast, wing, thigh, and drumstick weights than those with NB (*P* < 0.05; Table 1). In addition, strain 1 with WB had greater BW and carcass weight than strain 1 birds with NB (*P* < 0.05). All strains with WB had greater breast weights than those with NB (*P* < 0.05). As for the percentage, strain 5 with WB had a greater carcass yield than NB birds (*P* < 0.05). Strains 2-5 with WB had a greater breast yield than those with NB (*P* < 0.05). Strains 2 and 3 with WB had a smaller tender percentage than those with NB (*P* < 0.05). No other difference existed between birds with WB and NB with respect to processing yield.

### pH and Instrumental Color (CIE∗)

For birds with NB or WB, there were no differences among strains with respect to pH_15min_, *L∗*_24h_, *b∗*_24h_, hue angle, and chroma (*P* < 0.05) with the exception of pH_24h_ and *a∗*_24h_ for NB (Table 2). The NB of strain 5 had a higher pH_24h_ than strain 2 (*P* < 0.05) but did not differ from other strains (*P* > 0.05). The NB of strain 1 had a greater *a∗*_24h_ than strain 4 (*P* < 0.05) but did not differ from other strains (*P* > 0.05) based on the mean separation results (Table 2). Within each strain, strain 5 WB had a higher pH_15min_ than NB (*P* < 0.05) and strain 2 WB had a higher pH_24h_ than NB (*P* < 0.05). With respect to color, strain 4 WB had greater *a∗*_24h_ and chroma than NB (*P* < 0.05), and strain 3 WB had greater *b∗*_24h_ and chroma than NB (*P* < 0.05) (Table 2).

**Table 2:** pH, color, cooking loss, and shear force measurements of normal and woody breast from 5 strains of broilers.

Strain (N)^1^	pH_15min_	pH_24h_	*L∗*_24h_	*a∗*_24h_	*b∗*_24h_	Hue	Chroma	Cooking loss (%)^3^	Shear force (N)^3^
Birds with Normal Breast									
Strain 1 (12)	6.50	5.83^a,b^	62.8	8.49^a^	17.8	64.3	19.8	25.2	18.3^a^
Strain 2 (10)	6.52	5.73^b,^∗	64.2	7.63^a,b^	16.7	65.5	18.4	24.1	16.4^a,b^
Strain 3 (17)	6.44	5.79^a,b^	63.8	7.47^a,b^	16.6∗	65.9	18.2∗	27.3	14.7^b^
Strain 4 (17)	6.44	5.83^a,b^	63.6	6.98^b,^∗	16.5	66.9	17.9∗	24.0	18.0^a^
Strain 5 (4)	6.34∗	5.97^a^	63.3	7.74^a,b^	16.3	64.5	18.1	25.3	15.8^a,b^
SEM	0.067	0.046	0.944	0.473	0.775	1.397	0.787	1.98	1.056
*P*-value	0.562	0.058^2^	0.818	0.100^2^	0.608	0.530	0.316	0.533	0.013
Birds with Woody Breast									
Strain 1 (19)	6.48	5.86	64.2	8.24	18.0	65.4	19.8	27.0	17.4^a,b^
Strain 2 (22)	6.54	5.87∗	64.9	8.20	18.0	65.5	19.8	25.0	15.9^b^
Strain 3 (15)	6.51	5.86	65.4	8.65	18.9∗	65.5	20.8∗	28.8	16.3^a,b^
Strain 4 (14)	6.54	5.90	64.5	8.06∗	18.1	66.0	19.9∗	20.0	18.7^a,b^
Strain 5 (28)	6.52∗	5.95	64.2	8.14	17.5	65.2	19.4	27.6	18.8^a^
SEM	0.038	0.038	0.721	0.355	0.479	0.819	0.526	2.001	0.872
*P*-value	0.844	0.245	0.733	0.840	0.376	0.977	0.392	0.076	0.030

^a-b^Means with the same letter are not different due to strain within normal or woody breast bird groups.

Means without a ‘∗’ are not different between normal and woody breast birds within each strain.

1 For each strain, 32 birds were selected for processing. Strains 1 and 4 had 31 samples due to the missing tags in the processing.

2 ANOVA test did not show significance due to the very small sample size of strain 5 in NB group (n = 4) that does not provide enough power to show significant results. Power is the probability of rejecting the null hypothesis when the alternative hypothesis is true.

3 For each strain, only half of the chicken breast samples (2 birds/pen, n = 16 in total) were measured for cooking loss and shear force.

### Relationship Between WB Severity and Other Attributes

Woody breast score was positively correlated with WS, breast weight and breast yield for each strain (*P* < 0.05) (Table 3). Body weight was found to be positively correlated with WB within strains 2, 3 and 5 (*P* < 0.05). However, WB was only positively associated with pH_24h_ within strain 2 (*P* = 0.001).

**Table 3:** The coefficients and *P* value of Spearman's rank correlation of WB score with white striping, pH_24h_, BW, breast weight and yield of 5 strains of broiler fed a control diet.

Strain	White striping		pH_24h_		BW		Breast weight		Breast yield	
	Coefficient	*P*-value	Coefficient	*P*-value	Coefficient	*P*-value	Coefficient	*P*-value	Coefficient	*P*-value
Strain 1	0.395	0.030	0.103	0.594	0.308	0.098	0.413	0.023	0.452	0.014
Strain 2	0.352	0.048	0.545	0.001	0.353	0.048	0.523	0.002	0.502	0.003
Strain 3	0.557	0.001	0.206	0.258	0.507	0.003	0.631	0.0001	0.497	0.004
Strain 4	0.492	0.005	0.198	0.285	0.289	0.115	0.410	0.022	0.404	0.024
Strain 5	0.547	0.001	0.164	0.369	0.552	0.001	0.666	<0.0001	0.503	0.004

### Cooking Loss and Shear Force

There were no differences in cooking loss within birds with NB (*P* = 0.533) or WB (*P* = 0.076) (Table 2). There were also no differences between NB and WB of any strains (*P* > 0.05) (Table 2). Within NB, strains 1 and 4 had greater shear force than strain 3 (*P* = 0.013), no other difference existed (*P* > 0.05). Within WB, strain 5 had a greater shear force than strain 2 (*P* = 0.030) but did not differ from other strains (*P* < 0.05) (Table 2). There were no differences in shear force between NB and WB of any broiler strains (*P* > 0.05) (Table 2).

### Proteins that Were Differentially Expressed Between Strains

Protein profiles of NB and WB samples were evaluated among strains and presented in Tables 4 and 5. These identified proteins belong to different functional groups and are listed in the following paragraphs.

**Table 4:** Identification of differentially expressed proteins in normal breast from broilers of different strains.

Proteins (strains A/B)	Protein ID	Gene	Coverage (%)	MW (kDa)	Calc. pI	A/B fold change^1^	Over-abundance in	Category	Function^2^
Strain 5/Strain 2									
Actin, alpha skeletal muscle	P68139	ACTA1	70.0	42.0	5.39	2.29	Strain 5	Contractile	Actin and actin related protein
Cofilin-2, muscle isoform	17433708	CFL2	94.6	18.6	7.88	2.17	Strain 5	Structural	Non-motor actin binding protein
Ubiquitin carboxyl-terminal hydrolase	A1IMF0	UCH-L1	60.5	25.1	6.07	2.06	Strain 5	Enzymatic	Cysteine protease
Voltage-dependent anion-selective channel protein 2 isoform X1	XP_015143678.1	VDAC2	66.6	31.5	8.63	3.25	Strain 5	Transport	Voltage-gated ion channel
Strain 4/Strain 3									
Myozenin-3	118097461	MYOZ3	84.2	26.7	7.03	2.45	Strain 4	Structural	Non-motor actin binding protein
Strain 5/Strain 3									
Nucleoside diphosphate kinase	45384260	NDPK	92.2	17.3	7.90	2.01	Strain 5	Enzymatic	NF^3^
Voltage-dependent anion-selective channel protein 2 isoform X1	XP_015143678.1	VDAC2	62.8	31.5	8.63	5.06	Strain 5	Transport	Voltage-gated ion channel
Strain 5/Strain 4									
Myozenin-1 isoform X2	XP_004942140.1	MYOZ1	90.3	29.5	8.12	2.12	Strain 5	Structural	NF

1 Comparison ratio was the protein expression of A/B (ratio > 2.0 or ratio < 0.5).

2 Functions of protein were found on the PANTHER (protein annotation through evolutionary relationship) classification system against *Gallus gallus*.

3 NF: Proteins were not found on the PANTHER.

**Table 5:** Identification of differentially expressed proteins in woody breast from broilers of different strains.

Proteins (strains A/B)	Protein ID	Gene	Coverage (%)	MW (kDa)	Calc. pI	A/B fold change^1^	Over-expressed in	Category	Function^2^
Strain 3/Strain 1									
Pyruvate kinase	P00548	PKM	72.8	58.0	7.61	2.22	Strain 1	Enzymatic	Kinase
Strain 4/Strain 1									
PIT54 protein precursor	46395491	PIT54	63.4	50.8	4.73	0.31	Strain 1	Receptor	NF^3^
Strain 5/Strain 1									
Alpha-actinin 2	P20111	ACTN2	60.8	104.2	5.39	0.38	Strain 1	Structural	Calponin homology domain-containing protein
Elongation factor 2	Q90705	EEF2	75.3	95.3	6.83	2.22	Strain 5	Chaperone	Translation elongation
Gelsolin	O93510	GSN	59.0	85.8	6.32	0.44	Strain 1	Structural	Non-motor actin binding protein
Myozenin-3	118097461	MYOZ3	94.2	26.7	7.03	6.52	Strain 5	Structural	Non-motor actin binding protein
Strain 3/Strain 2									
Beta-enolase	P07322	ENO3	59.9	47.2	7.61	0.33	Strain 2	Enzymatic	Lyase
Strain 5/Strain 2									
Adenosylhomocysteinase	971428595	AHCY	92.2	47.7	6.89	2.92	Strain 5	Enzymatic	Hydrolase
LIM domain-binding protein 3	NP_001273190.1	LDB3	70.0	31.2	9.32	4.05	Strain 5	Structural	Actin-binding cytoskeletal protein
Strain 4/Strain 3									
Alpha-enolase	F1NZ78	ENO1	81.3	47.3	6.80	0.45	Strain 3	Enzymatic	Lyase
Strain 5/Strain 3									
LIM domain-binding protein 3	NP_001273190.1	LDB3	67.8	31.2	9.32	2.92	Strain 5	Structural	Actin-binding cytoskeletal protein
Myozenin-3	118097461	MYOZ3	84.2	26.7	7.03	7.49	Strain 5	Structural	Non-motor actin binding protein
Strain 5/Strain 4									
Actin-related protein 3	Q90WD0	ACTR3	60.1	47.4	5.88	21.9	Strain 5	Contractile	Actin related protein
Annexin A5	P17153	ANXA5	85.1	36.2	5.82	2.10	Strain 5	Transport	Calcium-binding protein
Desmin	XP_015145578.2	DES	86.6	53.5	5.38	2.67	Strain 5	Structural	Glial fibrillary acidic protein
60 kDa heat shock protein, mitochondrial	Q5ZL72	HSP60	83.1	60.9	5.87	0.29	Strain 4	Chaperone	Unfolded protein binding
Myosin-binding protein H	Q05623	MYBPH	62.0	58.6	7.53	0.46	Strain 4	Contractile	Myosin-binding protein
Serum albumin precursor	NP_990592.2	ALB	87.6	69.8	5.74	2.91	Strain 5	Transport	Transfer/carrier protein
Troponin T, fast skeletal muscle isoform	O57559	TNNT3	44.9	33.8	7.09	2.59	Strain 5	Contractile	Actin binding motor protein
Ubiquitin carboxyl-terminal hydrolase	A1IMF0	UCH-L1	63.0	25.1	6.07	2.08	Strain 5	Enzymatic	Cysteine protease

1 Comparison ratio was the protein expression of A/B (ratio > 2.0 or ratio < 0.5).

2 Functions of protein were found on the PANTHER (protein annotation through evolutionary relationship) classification system against *Gallus gallus*.

3 NF: Proteins were not found on the PANTHER.

#### Structural Proteins

A few structural proteins were more abundant in strain 5 compared to other strains of broilers. Cofilin-2 (**CFL2**) was more abundant in strain 5 NB compared to strain 2 (*P* < 0.05) (Table 4). Myozenin-1 isoform X2 (**MYOZ1**) in NB and desmin (**DES**) in WB were more abundant in strain 5 compared to strain 4 NB and WB, respectively (*P* < 0.05) (Tables 4 and 5). Myozenin-3 (**MYOZ3**) was more abundant in strain 5 WB compared to strains 1 and 3 (*P* < 0.05). LIM domain-binding protein 3 (**LDB3**) was more abundant in WB of strain 5 compared to strains 2 and 3 (*P* < 0.05). In addition, strain 5 WB was less abundant in alpha-actinin 2 (**ACTN2**) and gelsolin (**GSN**) than strain 1 WB (*P* < 0.05). The only other difference in strains for structural proteins was that of a higher abundance of MYOZ3 in strain 4 NB compared to strain 3 NB (*P* < 0.05).

#### Contractile Proteins

Differences in contractile proteins were present between strain 5 and strains 4 and 2 (Tables 4 and 5). Within NB, actin, alpha skeletal muscle (**ACTA1**) was more abundant in strain 5 than strain 2 (*P* < 0.05). Within WB, actin-related protein 3 (**ACTR3**) and troponin T, fast skeletal muscle isoform (**TNNT3**) were more abundant in strain 5 than strain 4 (*P* < 0.05), and myosin-binding protein H (**MYBPH**) was less abundant in strain 5 than strain 4 (*P* < 0.05).

#### Enzymatic Proteins

Within NB, ubiquitin carboxyl-terminal hydrolase (**UCH-L1**) and nucleoside diphosphate kinase (**NDPK**) were more abundant in strain 5 compared to strain 2 and strain 3 (*P* < 0.05) (Table 4). Within WB, adenosylhomocysteinase (**AHCY**) and UCH-L1 were more abundant in strain 5 compared to strains 2 (*P* < 0.05) and strain 4 (*P* < 0.05), respectively (Table 5). The abundances of pyruvate kinase (**PKM**), beta-enolase (**ENO3**), and alpha-enolase (**ENO1**) in strain 3 WB were different from strains 1, 2 and 4, respectively (*P* < 0.05) (Table 5).

#### Transport Proteins

Differences in transport proteins were present between strain 5 and strains 2, 3 and 4. Within NB, voltage-dependent anion-selective channel protein 2 isoform X1 (**VDAC2**) was more abundant in strain 5 than strains 2 and 3 (*P* < 0.05) (Table 4). Within WB, annexin A5 (**ANXA5**), and serum albumin precursor (**ALB** precursor) were more abundant in strain 5 than strain 4 (*P* < 0.05) (Table 5).

#### Chaperones and Others

Elongation factor 2 (**EEF2**) was more abundant in strain 5 WB compared to strain 1 (*P* < 0.05), and mitochondrial 60 kDa heat shock protein (**HSP60**) was less abundant in strain 5 WB compared to strain 4 (*P* < 0.05) (Table 5). In strain 4 WB, PIT54 protein precursor (**PIT54**) was less abundant compared to strain 1 (*P* < 0.05) (Table 5).

## Discussion

### Growth Performance and Breast Quality of Different Strains of Broilers

The WB myopathy is related to the selection of broiler birds with increased growth rate, carcass weight and breast yield. In the current study, broilers of strains 1-5 at 55 d of age had averaged BWs of 3.88, 3.86, 4.25, 4.23 and 3.99 kg, respectively (Zhang et al., 2020b), which fall in the target BW ranges of commercial broilers with strains 1-4 being at 3.6 to 4.5 kg and strain 5 being at 3.6 to 4.7 kg. These 5 genetic strains of broilers that are all fast-growing birds with averaged for strain yielded WB percentages (moderate and severe WB) from 45% for strain 4 to 88% for strain 5 when the birds were fed with the same diet and raised in the same environment, indicating a variation in WB incidence among genetic lines. The processing weight and yield data also support this statement. For example, strain 2 birds with WB were not different from strain 2 birds with NB (*P* > 0.05) but weighed less than strain 3 and strain 4 with NB (Table 1). In addition, birds of strain 3 were heavier than strain 5 in both NB and WB group, but the WB incidence of strain 3 was much lower than strain 5 (Table 1). These facts support the genetic basis of WB development but also suggest that the BW at harvest is not a primary determinant for WB myopathy for all genetic strains even though it is generally accepted that heavier birds tend to have WB within each strain. It has been suggested that WB myopathy may appear as early as 14 d (Radaelli et al., 2017). On day 14, strain 3 birds (n = 128) were heavier (*P* < 0.05) than other strains (n = 128/strain) that were not different (*P* > 0.05) from each other (Zhang et al., 2020b), which indicates that BW at the early life of birds is not a determinant for WB myopathy either. In comparison, WB is associated with breast weight because birds with WB had heavier breast weights than those with NB for all 5 strains, which agrees with a previous study conducted by Sihvo et al. (2014) in which they concluded that increased breast muscle weight is a key factor in WB muscle myopathy. Birds with WB showed genetic variations on all weights but breast and tender, suggesting that the bird would likely develop WB if the breast was too heavy (>830 g on average) regardless of the genetic strains. Birds with WB also showed genetic variations in all processing yields but the wing. The breast yield of strain 5 with WB was greater than strains 1, 3, and 4 with WB but did not differ from strain 2. There was a 4.4% increase in breast yield of strain 5 with WB compared to strain 5 with NB but only 1.1 to 1.4% increase for other strains. Breast yield results concur with Kuttappan et al. (2017a), who determined that severe WB had a greater breast yield than NB meat. Therefore, the WB development and incidence are not only related to breast weight and yield but also genetics. Hubert et al. (2018) suggested a mechanistic, heritable basis for WB after comparing the transcriptome data between commercial fast-growth and slow-growth broiler strains. Alnahhas et al. (2016) observed a positive genetic association between WS and muscle ultimate pH, and suggested that genetics is major determinant of WS. The genetic basis was also investigated for WS through the identification of quantitative traits loci in high-yield broilers with results suggesting a polygenic inheritance of the WS defect (Pampouille et al., 2018). These 2 aforementioned research may also support the existence of a genetic basis for WB since WB and WS share common characteristics (Griffin et al., 2018). However, the Pampouille et al. (2018) study may not be applicable to commercial broilers since it was conducted on an inbred stain and can therefore overestimate genetic contributions. In contrast, a previous study compared the differences in selection history for increased breast yield between 2 purebred commercial broiler lines with breast yields of 21% and 29%, the results indicated a very low heritability (the ratio between the additive genetic variance and the phenotypic variance) for WB, which suggests a non-genetic basis explaining the variation in WB incidence (Bailey et al., 2015). A similar conclusion was drawn in another study conducted by the same group with a pure-bred commercial broiler line (Bailey et al., 2020). The inconsistent results may be due to the differences in the sample size, the genetic lines that were used, the technique used to evaluate the genetic contribution, etc.

Normal pH values of chicken breast at 15 min postmortem are 6.20-6.50 (Berri et al., 2005). In this study, pH_15min_ values were 6.34-6.54. The absolute average pH_15min_ values for WB of strains 2-5 and NB of strain 2 were greater than 6.50, indicating a slight increase in pH for modern fast-growing broilers. Normal ultimate pH (pH_24h_) values are 5.80-5.90 (Petracci et al., 2017; Baldi et al., 2020) and in the current study pH_24_ values ranged from 5.73 to 5.97. The pH was measured using a penetration pH probe on the cranial part of the chicken breast where the WB myopathy was often observed, therefore we observed relatively higher pH values. The absolute values of pHu of WB were numerically greater than NB for strains 1-4 (Table 2). Also, WB had higher pH_15min_ (*P* = 0.0474) and pHu (*P* = 0.0014) when averaged over broiler strains. As for color measurements, although our results were not consistent across all strains, the absolute differences between NB and WB of strains 2-5 are consistent with previous literature that WB is redder and yellower than NB meat (Dalle Zotte et al., 2017; Cai et al., 2018). Although multiple literatures reported that WB had a greater cooking loss than NB meat (Dalle Zotte et al., 2017; Dalgaard et al., 2018), this was not observed in the current study. The tenderness among meat samples varies due to animal age, strain, sex, deboning time, aging time, environmental stress, and feeds. These factors affect the mass of muscle fibers and the percentage of connective tissue in muscle (Ismail and Joo, 2017). In the present study, strain variation was found in the shear force of cooked NB where strain 3 was more tender than strains 1 and 4, and cooked WB where strain 2 was more tender than strain 5 (Table 2), which might be due to the connective tissue content and the level of proteolysis of myofibrillar proteins during post-mortem storage (Marcinkowska-Lesiak et al., 2016). However, no difference existed between NB and WB within each strain (Table 2). Shear force results for WB have been contradictory, and therefore may not accurately depict WB from NB. In addition, the shear force values were between 14.7 and 18.8 N, far below 45 N, a cutoff number that consumers evaluated chicken meat as tender or tough (Schilling et al., 2008). It has been found in many studies, however, that WB was less tender than NB in texture profile analysis and descriptive sensory analysis (Dalgaard et al., 2018; Jarvis et al., 2020).

### Protein Expression of Normal Chicken Breast From Different Broiler Strains

The effects of genetics on NB muscle proteome profiles were limited to small changes in the abundance of 7 proteins, including 1 contractile, 3 structural, 2 enzymatic and 1 transport proteins (Table 4). Normal breast muscle protein profiles in strains 1-4 showed large similarities with only one protein (MYOZ3) that was differentially expressed between strains 3 and 4 (Table 4). Strain 5 NB muscle was most different from others, with 4, 2 and 1 proteins that were expressed differently compared to strains 2, 3 and 4, respectively (Table 4). First, MYOZ1 was more abundant in strain 5 than strain 4. Frey and Olson (2002) reported that MYOZ1 negatively regulates skeletal muscle tissue regeneration and development, which may partially explain the smaller BW and averaged absolute breast weight of strain 5 in comparison to strain 4. In addition, another structural protein, CFL2, and one contractile protein, ACTA1, were differentially expressed in NB between strains 5 and 2. Cofilin-2 belongs to the actin depolymerization factor family and regulates actin filament dynamics (Kanellos and Frame, 2016) while ACTA1 interacts with other proteins, specifically myosin, to produce force for muscle contraction. Thus, the decreased CFL2 and ACTA1 may indicate a weak muscle structure with decreased contractibility in strain 2 birds (Balakrishnan et al., 2019). Moreover, strain 5 NB had a higher abundance of VDAC2 compared to strains 2 and 3. It is known that VDAC2 mediates the flow of ions and metabolites across the outer mitochondria membrane and allow the movement of superoxide from mitochondria to cytosol (Han et al., 2003). Overexpression of VDAC2 may increase the oxidative reactions of enzymes and others in cell cytosol, implying that strain 5 NB muscle cells may be more oxidative than those from strains 2 and 3. VDAC2 is also involved in the mitochondrial recruitment of BAK, a protein from the BCL2 protein family, that is responsible for inducing apoptosis (Veresov and Davidovskii, 2014). The combined effect of oxidative stress and increased apoptosis, the known causes of WB myopathy (Hubert et al., 2018), might indicate that strain 5 is genetically more prone to develop WB since 28 out of 32 birds of strain 5 developed WB (Table 1). Finally, 2 enzymes (UCH-L1 and NDPK) were more abundant in NB from strain 5 in comparison to strains 2 and 3 (Table 4). The upregulated UCH-L1 in strain 5 NB promotes myoblast activation and proliferation during muscle regeneration and repair processes (Gao et al., 2017), while an increased NDPK may indicate the upregulation of DNA and protein synthesis in strain 5 NB because NDPK is an ubiquitous enzyme that catalyzes the transfer of the γ -phosphate of a (deoxy)nucleoside triphosphate to a (deoxy) nucleoside diphosphate, and processes transcriptional regulation and protein histidine kinase activities (Roymans et al., 2002).

### Protein Expression of Woody Chicken Breast From Different Broiler Strains

For WB, 20 proteins exhibited differential abundance between strains. Only 2 out of 10 comparisons among 5 strains did not show any differences in WB protein profiles, including strains 1 vs. 2 and strains 2 vs. 4. No differences in NB protein profiles were found for these 2 pairs of comparisons either. It is not surprising to see the similarity in breast proteomes of strains 1 and 2 since they share a similar genetic background and did not show any phenotypic differences in processing weight, processing yield, pH or color attributes within NB or WB group. However, strains 2 and 4 are from different genetic backgrounds and differed in BW and breast yield within NB and WB groups. Therefore, genetics is one contributing factor to muscle development and meat quality since the proteome changes during animal growth as a response to nutrition, management, stress, etc. Strain 5 exhibited differences from all other strains in the expression of structural, contractile, enzymatic, transport, and chaperone proteins (Table 5).

Sarcomeres are composed of 4 structural elements, including Z-discs (e.g. ACTN2, LDB3, and MYOZ3), thick myosin-containing filaments (e.g. MYBPH), thin actin-containing filaments (e.g. ACTR3, TNNT3, GSN), and intermediate filaments (e.g. DES). The increased abundance in MYOZ3 in strain 5 WB compared to strains 2 and 3 WB indicates that strain 5 WB may have a greater muscle growth rate since MYOZ3 plays a role in cell proliferation (Ye et al., 2017), which is consistent with a higher breast weight and breast yield in strain 5. Both MYOZ3 and LDB3 contribute to Z-disc formation. The lower abundance of any of these 2 proteins in WB of strains 2 and 3 compared to strain 5 may lead to aberrant Z-disc signal transduction and muscle development (Ye et al., 2017). Another Z-disc protein, ACTN2 plays a role in thin filament organization and the interaction between muscle membrane and the sarcomeric cytoskeleton (Mills et al., 2001). Gelsolin is a Ca^2+^ regulated actin-binding protein that can sever and cap the filament servers and nucleate actin polymerization (Hlushchenko and Hotulainen, 2019). The higher abundance of MYOZ3 in combination with a lower abundance of ACTN2 and GSN may suggest an unbalanced protein interaction and a weakening Z-disc and cytoskeleton structure in strain 5 WB. One structural and 3 contractile proteins were differentially present in WB of strains 4 and 5, 2 broiler strains of similar genetic background, indicating a potential difference in their muscle structure and contractibility. Desmin, the largest intermediate filament, maintains cell integrity by connecting Z-discs to the sarcolemmal cytoskeleton as well as nucleus and mitochondria, which strengthens the muscles when used (Hnia et al., 2015). Human DES-related skeletal and cardiac myopathies are characterized by abnormal accumulation of DES within muscle fibers (Goebel, 1995). The contractile protein MYBPH that is known to be specifically expressed in fast-twitch glycolytic muscle fibers showed lower abundance in strain 5 WB muscle, indicating muscle weakness since MYBPH maintains the structural integrity of the muscle (Hundley et al., 2006). Troponin-T is involved in the calcium-dependent regulation of skeletal muscle contraction but not in the maintenance of the muscle structure (Huang et al., 2011). The higher abundance of TNNT3 in strain 5 WB muscle may indicate that strain 5 birds with WB are straining regularly to contract and relax their breast muscles, which may be due to the reduced supply of oxygen to the breast muscle. This again supports that strain 5 birds might be more prone to develop WB.

Two enzymatic proteins, AHCY and UCH-L1, were more abundant in WB of strain 5 compared to strains 2 and 4, respectively (Table 5). Adenosylhomocysteinase converts s-adenosyl-homocysteine to homocysteine, which is either used in the transsulfuration pathway for glutathione synthesis or remethylated to methionine by methionine synthase (Sanderson et al., 2019). Therefore, the higher abundance of AHCY in strain 5 WB created more homocysteine and methionine, and increased methionine levels have been shown to increase BW in broilers (Esteve-Garcia and Mack, 2000), which may have been what happened for strain 5 in the current study in comparison to strain 2. This also suggests that strain 5 birds may have higher demand on methionine intake. When birds were fed with a reduced amino acid diet (the digestible amino acids, including lysine, methionine, cysteine and threonine, were 20% lower than the control diet), the birds of strain 5 were lighter but the birds of strain 2 remained the same BW when compared to birds that were fed the control diet (Zhang et al., 2020a), indicating a greater demand of digestible amino acids for strain 5 to maximize growth performance. Another enzyme, UCH-L1 is primarily expressed in neuronal tissue but upregulated in skeletal muscle in disease conditions, which affects skeletal muscle function through the neuro-control of muscles (Gao et al., 2017). It was also found that UCH-L1 was more abundant in WB than NB in strain 5 (Zhang et al., 2020a). Therefore, WB is not just a myopathy status in muscle but may be an indicator of neuronal tissue disease in affected birds.

Transport proteins, ANXA5 and ALB precursor, were also more abundant in strain 5 WB compared to strain 4 (Table 5). Annexin A5 is associated with transmembrane activity and collagen binding in the Ca^2+^ channel. Although the calcium concentration was not measured in the present study, Livingston (2018) observed an increased calcium accumulation in WB and WS tissue which may contribute to muscle contraction due to ante mortem rigor (Livingston, 2018). This may partially explain the severity of the WB stiffness and rigidity in strain 5 birds. Annexin A5 is also known to positively regulate the apoptotic process (Gaudet et al., 2011), which is a potential contributor to WB formation (Zhang et al., 2020a). Serum albumin is the most abundant serum protein whose redox modifications modulate its physiological function, as well as serves as a biomarker for oxidative stress (Prakash, 2017). Strain 5 birds may undergo more intense contraction and oxidative stress when compared to strain 4.

Three glycolytic enzymes, PKM, ENO3, and ENO1, were differently abundant in strain 3 WB compared to strains 1, 2 and 4, respectively (Table 5). Enolases (ENO1, ENO2 and ENO3) catalyze the dehydration of 2-phosphoglycerate to phosphoenolpyruvate, which is converted to pyruvate by PKM. Although broiler breast muscles are primarily glycolytic and use anaerobic glucose metabolism to produce energy (Lilburn et al., 2019), the differences in glycolytic enzymes between strains may indicate the differences in glycolytic activities among different strains. In severe WB, glycolysis and gluconeogenesis are the 2 most down-regulated pathways (Kuttappan et al., 2017b). The down regulation of glycolysis means that less energy was produced in the breast muscle, which contributes to a higher pH in WB. Alpha-enolase is a multifunctional protein, that is expressed differently under pathological stress, performing several of its multiple functions, mainly as a plasminogen receptor in muscle regeneration after injury by modulating pericellular fibrinolytic activity (Díaz-Ramos et al., 2012). Strain 3 birds with WB may be under more stress and therefore more active in muscle regeneration in comparison to strain 4 due to a higher abundance of ENO1 in strain 3 WB.

To deal with environmental stress, organisms use chaperone proteins to protect and stabilize the cellular proteome. Two chaperone proteins were identified in the current study. EEF2 was more abundant in WB of strain 5 compared to strain 1 and mitochondrial HSP60 that was less abundant in WB of strain 5 compared to strain 4 (Table 5). Elongation factor 2 is responsible for guanosine triphosphate-dependent ribosomal translocation during translation elongation, and therefore protein synthesis. The higher abundance of EEF2 may suggest a higher protein synthesis rate in strain 5 WB considering the higher breast yield of strain 5 than strain 1. Mitochondria HSP60 is predominantly present in mitochondria and is involved in the folding of proteins entering the mitochondria. The presence of high levels of HSP60 in the mitochondria protects mitochondrial proteins from unfolding and potentially prevents age-associated increases in ROS-production by mitochondrial proteins involved in oxidative reactions (Krivoruchko and Storey, 2010). Therefore, the lower abundance of HSP60 in WB suggests that strain 5 birds exhibited a weaker protection against protein unfolding and oxidative stress, which may explain the higher WB incidence of strain 5 compared with other strains.

## Conclusions

It was confirmed that the WB myopathy is closely related to increased breast weights. Also, genetic variation that causes phenotypic differences were observed in 5 strains of broilers that produce NB and WB. The proteomes of broiler breast muscle act as a molecular link between the genome and phenotypic characteristics. Strain 5 breast proteomes were most different from other strains in both NB and WB groups. Strain 5 birds are genetically more prone to develop WB when birds were fed the control diet due to the evidence of increased apoptosis and protein synthesis, more intense contraction, and high oxidative stress in strain 5 WB muscle in comparison to other strains. In the future, the genome sequencing of broiler strains will facilitate the application of proteomics to detect biomarkers for meat defects and monitoring the effectiveness of corrective strategies.
